# Supplementary material for: Structure of the Gq-coupled adhesion receptor ADGRL4
Source: Nat Commun. 2025 Dec 30;17:907. doi: 10.1038/s41467-025-67629-0 (PMC12830901; doi:10.1038/s41467-025-67629-0)
Supplement: Supplementary file 1 — Supplementary Information [file 41467_2025_67629_MOESM1_ESM.pdf]

Supplementary data

Supplementary Tables:

Proximity assay comparisons	Normalised luminescence	SEM	*p*-value	level of significance
FL1 + mini-G_q	10.9	0.8	0.003	**
FL1 + mini-$G_q\Delta\alpha5$	7.4	0.6		
FLmut1 + mini-G_q	9.2	0.8	0.05	ns
FLmut1 + mini-$G_q\Delta\alpha5$	7.2	0.5		
CTF1 + mini-G_q	16.6	0.7	<0.0001	****
CTF1 + mini-$G_q\Delta\alpha5$	6.3	0.3		
HCRTR2 + mini-G_q	32.2	1.3	0.5	ns
HCRTR2 + mini-$G_q\Delta\alpha5$	30.7	1.8		
HCRTR2 + orexin-a peptide + mini-G_q	179.1	12.2	<0.0001	****
HCRTR2 + orexin-a peptide + mini-$G_q\Delta\alpha5$	30.9	2.4		
FL1 + mini-G_q	10.9	0.8	0.1	ns
FLmut1 + mini-G_q	9.2	0.8		
FL1 + mini-G_q	10.9	0.8	<0.0001	****
CTF1 + mini-G_q	16.6	0.7		
FLmut1 + mini-G_q	9.2	0.8	<0.0001	****
CTF1 + mini-G_q	16.6	0.7		
FL1 + mini-G_s	8.1	0.3	<0.0001	****
FL1 + mini-$G_s\Delta\alpha5$	9.9	0.2		
FLmut1 + mini-G_s	8.0	0.5	0.008	**
FLmut1 + mini-$G_s\Delta\alpha5$	14.4	2.0		
CTF1 + mini-G_s	5.2	0.4	0.5	ns
CTF1 + mini-$G_s\Delta\alpha5$	5.8	0.8		
ADRB1 + mini-G_s	29.5	1.8	0.05	ns
ADRB1+ mini-$G_s\Delta\alpha5$	24.7	1.5		
ADRB1+ formoterol + mini-G_s	79.4	3.8	<0.0001	****
ADRB1+ formoterol + mini-$G_s\Delta\alpha5$	29.6	1.6		
FL1 + mini-G_s	8.1	0.3	0.8	ns
FLmut1 + mini-G_s	8.0	0.5		
FLmut1 + mini-G_s	8.0	0.5	0.0008	***
CTF1 + mini-G_s	5.2	0.4		
FL1 + mini-G_s	8.1	0.3	<0.0001	****
CTF1 + mini-G_s	5.2	0.4		
FL1 + mini-G_{12}	36.2	5.6	0.5	ns
FL1 + mini-$G_{12}\Delta\alpha5$	42.8	6.6		
FLmut1 + mini-G_{12}	37.6	5.5	0.7	ns
FLmut1 + mini-$G_{12}\Delta\alpha5$	40.0	4.9		

CTF1 + mini-G_{12}	20.5	2.4	0.3	ns
CTF1 + mini-$G_{12}\Delta\alpha5$	24.9	3.1		
GPR35 + mini-G_{12}	72.8	2.2	<0.0001	****
GPR35 + mini-$G_{12}\Delta\alpha5$	49.6	2.4		
GPR35 + Lodoxamide + mini-G_{12}	757.2	91.1	<0.0001	****
GPR35 + Lodoxamide + mini-$G_{12}\Delta\alpha5$	49.4	2.7		
FL1 + mini-G_{12}	36.2	5.6	0.9	ns
FLmut1 + mini-G_{12}	37.6	5.5		
FLmut1 + mini-G_{12}	37.6	5.5	0.01	*
CTF1 + mini-G_{12}	20.5	2.4		
FL1 + mini-G_{12}	36.2	5.6	0.02	*
CTF1 + mini-G_{12}	20.5	2.4		
FL1 + mini-G_o	1.1	0.1	<0.0001	****
FL1 + mini-$G_o\Delta\alpha5$	0.6	0.02		
FLmut1 + mini-G_o	1.1	0.1	0.004	**
FLmut1 + mini-$G_o\Delta\alpha5$	0.7	0.07		
CTF1 + mini-G_o	0.7	0.06	0.003	**
CTF1 + mini-$G_o\Delta\alpha5$	0.4	0.04		
APLNR + mini-G_o	1.3	0.2	0.9	ns
APLNR + mini-$G_o\Delta\alpha5$	1.3	0.08		
APLNR + CMF-019 + mini-G_o	6.1	1	0.0002	***
APLNR + CMF-019 + mini-$G_o\Delta\alpha5$	1.2	0.1		
FL1 + mini-G_o	1.1	0.1	0.6	ns
FLmut1 + mini-G_o	1.1	0.1		
FLmut1 + mini-G_o	1.1	0.1	0.004	**
CTF1 + mini-G_o	0.7	0.06		
FL1 + mini-G_o	1.1	0.1	0.0006	***
CTF1 + mini-G_o	0.7	0.06		
FL1 + β-arrestin 1	7.6	0.8	0.8	ns
FLmut1 + β-arrestin 1	7.3	0.7		
FLmut1 + β-arrestin 1	7.3	0.7	0.0003	***
CTF1 + β-arrestin 1	3.8	0.2		
FL1 + β-arrestin 1	7.6	0.8	0.0004	***
CTF1 + β-arrestin 1	3.8	0.2		
FL1 + β-arrestin 2	28.6	4.2	0.5	ns
FLmut1 + β-arrestin 2	33.1	5.2		
FLmut1 + β-arrestin 2	33.1	5.2	0.02	*
CTF1 + β-arrestin 2	17.6	2.2		
FL1 + β-arrestin 2	28.6	4.2	0.03	*
CTF1 + β-arrestin 2	17.6	2.2		
**Proximity assay comparisons**	**Percentage of HA-positive**	**SEM**	***p*-value**	**level of significance**

	**receptor normalised luminescence**			
FL1: (FL1+mini-G_q) - (FL1+mini-$G_q\Delta\alpha5$)	100.0%	23.9%	0.0003	***
ΔHA-FL1: (ΔHA-FL1+mini-G_q) - (ΔHA-FL1+mini-$G_q\Delta\alpha5$)	321.1%	32.5%		
FLmut1: (FLmut1+mini-G_q) - (FLmut1+mini-$G_q\Delta\alpha5$)	100.0%	22.7%	0.9	ns
ΔHA-FLmut1: (ΔHA-FLmut1+mini-G_q) - (ΔHA-FLmut1+mini-$G_q\Delta\alpha5$)	98.0%	20.7%		
CTF1: (CTF1+mini-G_q) - (CTF1+mini-$G_q\Delta\alpha5$)	100.0%	15.7%	<0.0001	****
ΔHA-CTF1: (ΔHA-CTF1+mini-G_q) - (ΔHA-CTF1+ mini-$G_q\Delta\alpha5$)	369.8%	35.9%		
FL1: (FL1+mini-G_o) - (FL1+mini-$G_o\Delta\alpha5$)	100.0%	8.5%	0.2	ns
ΔHA-FL1: (ΔHA-FL1+mini-G_o) - (ΔHA-FL1+mini-$G_o\Delta\alpha5$)	114.4%	7.7%		
FLmut1: (FLmut1+mini-G_o) - (FLmut1+mini-$G_o\Delta\alpha5$)	100.0%	6.9%	0.9	ns
ΔHA-FLmut1: (ΔHA-FLmut1+mini-G_o) - (ΔHA-FLmut1+mini-$G_o\Delta\alpha5$)	101.4%	7.6		
CTF1: (CTF1+mini-G_o) - (CTF1+mini-$G_o\Delta\alpha5$)	100.0%	12.1%	0.1	ns
ΔHA-CTF1: (ΔHA-CTF1+mini-G_o) - (ΔHA-CTF1+ mini-$G_o\Delta\alpha5$)	78.1%	4.0%		

Supplementary Table 1 | **ADGRL4 couples weakly to mini-G_q.** (ns=not significant)

**Data collection and processing**	
Magnification	120,000x
Voltage (kV)	300
Electron exposure (e^-/Å^2)	60
Defocus range (μm)	-0.8 to -1.8
Pixel size (Å)	0.67
Symmetry imposed	-
Map resolution (Å)	3.14
FSC threshold	0.143
**Refinement**	
Model composition	
Non-hydrogen atoms	6756
Protein residues	870
R.m.s. deviations	
Bond lengths (Å)	0.003
Bond angles (°)	0.537
Validation	
MolProbity score	1.29
Clash score	5.35
Poor rotamers (%)	0
Ramachandran plot	
Favoured (%)	98.12
Allowed (%)	1.88
Disallowed (%)	0

Supplementary Table 2 | **Cryo-EM data collection, refinement and validation statistics**

Proximity assay comparisons	Percentage of CTF1 Normalised luminescence	SEM	*p*-value	level of significance
T407A: (T407A+ mini-G_q) - (T407A + mini-$G_q\Delta\alpha5$)	127.8%	9.9%	0.02	*
H408A: (H408A+ mini-G_q) - (H408A + mini-$G_q\Delta\alpha5$)	27.9%	2.6%	<0.0001	****
F409A: (F409A+ mini-G_q) - (F409A + mini-$G_q\Delta\alpha5$)	12.0%	1.0%	<0.0001	****
Ile411A: (Ile411A+ mini-G_q) - (Ile411A + mini-$G_q\Delta\alpha5$)	29.5%	4.7%	<0.0001	****
L412A: (L412A+ mini-G_q) - (L412A + mini-$G_q\Delta\alpha5$)	44.4%	2.1%	<0.0001	****
M413A: (M413A+ mini-G_q) - (M413A + mini-$G_q\Delta\alpha5$)	5.2%	5.3%	<0.0001	****
S414A: (S414A+ mini-G_q) - (S414A + mini-$G_q\Delta\alpha5$)	47.5%	5.4%	<0.0001	****
S415A: (S415A+ mini-G_q) - (S415A + mini-$G_q\Delta\alpha5$)	52.5%	5.2%	<0.0001	****

Proximity assay comparisons	Normalised luminescence	SEM	*p*-value	level of significance
CTF1 + mini-G_q	34.5	1.0	<0.0001	****
CTF1 + mini-$G_q\Delta\alpha5$	12.1	0.3		
T407A+ mini-G_q	39.8	2.1	<0.0001	****
T407A+ mini-$G_q\Delta\alpha5$	12.2	0.3		
H408A+ mini-G_q	21.0	0.4	<0.0001	****
H408A+ mini-$G_q\Delta\alpha5$	14.8	0.6		
F409A+ mini-G_q	16.8	0.3	<0.0001	****
F409A + mini-$G_q\Delta\alpha5$	14.1	0.3		
Ile411A+ mini-G_q	21.1	1.1	<0.0001	****
Ile411A + mini-$G_q\Delta\alpha5$	14.5	0.4		
L412A+ mini-G	22.0	0.4	<0.0001	****
L412A + mini-$G_q\Delta\alpha5$	12.1	0.2		
M413A+ mini-G_q	17.1	0.6	0.2	ns
M413A + mini-$G_q\Delta\alpha5$	15.9	0.8		
S414A+ mini-G_q	26.3	1.2	<0.0001	****
S414A + mini-$G_q\Delta\alpha5$	15.6	0.6		
S415A+ mini-G_q	25.7	1.0	<0.0001	****
S415A + mini-$G_q\Delta\alpha5$	14.1	0.4		

Proximity assay comparisons	Normalised luminescence	SEM	*p*-value	level of significance
CTF1 + mini-G_q	34.5	1.0	0.03	*
T407A+ mini-G_q	39.8	2.1		
CTF1 + mini-G_q	34.5	1.0	<0.0001	****
H408A+ mini-G_q	21.0	0.4		
CTF1 + mini-G_q	34.5	1.0	<0.0001	****
F409A+ mini-G_q	16.8	0.3		
CTF1 + mini-G_q	34.5	1.0	<0.0001	****
Ile411A+ mini-G_q	21.1	1.1		
CTF1 + mini-G_q	34.5	1.0	<0.0001	****
L412A+ mini-G	22.0	0.4		

CTF1 + mini-G_q	34.5	1.0	<0.0001	****
M413A+ mini-G_q	17.1	0.6		
CTF1 + mini-G_q	34.5	1.0	<0.0001	****
S414A+ mini-G_q	26.3	1.2		
CTF1 + mini-G_q	34.5	1.0	<0.0001	****
S415A+ mini-G_q	25.7	1.0		

Supplementary Table 3 | **Tethered agonist alanine mutagenesis mini-G_q coupling results.**

**Proximity assay comparisons**	**Percentage of CTF1 Normalised luminescence**	**SEM**	***p*-value**	**level of significance**
F505A: (F505A+ mini-G_q) - (F505A + mini-$G_q\Delta\alpha5$)	4.9%	2.7%	<0.0001	****
M508: (M508+ mini-G_q) - (M508+ mini-$G_q\Delta\alpha5$)	-19.7%	3.8%	<0.0001	****
W631A: (W631A + mini-G_q) - (W631A + mini-$G_q\Delta\alpha5$)	-7.9%	5.6%	<0.0001	****
Q656A: (Q656A+ mini-G_q) - (Q656A + mini-$G_q\Delta\alpha5$)	-5.8%	3.3%	<0.0001	****
F625A: (F625A + mini-G_q) - (F625A + mini-$G_q\Delta\alpha5$)	23.9%	7.7%	<0.0001	****
L626A: (L626A + mini-G_q) - (L626A + mini-$G_q\Delta\alpha5$)	25.5%	4.9%	<0.0001	****
L627A: (L627A + mini-G_q) - (L627A + mini-$G_q\Delta\alpha5$)	6.3%	9.6%	<0.0001	****
G628A: (G628A + mini-G_q) - (G628A + mini-$G_q\Delta\alpha5$)	0.3%	5.7%	<0.0001	****
H514A: (H514A + mini-G_q) - (H514A + mini-$G_q\Delta\alpha5$)	34.0%	4.5%	<0.0001	****
L515A: (L515A+ mini-G_q) - (L515A + mini-$G_q\Delta\alpha5$)	10.0%	5.0%	<0.0001	****
Y516A: (Y516A+ mini-G_q) - (Y516A + mini-$G_q\Delta\alpha5$)	5.8%	4.8%	<0.0001	****

**Proximity assay comparisons**	**Normalised luminescence**	**SEM**	***p*-value**	**level of significance**
CTF1 + mini-G_q	18.28	1.0	<0.0001	****
CTF1 + mini-$G_q\Delta\alpha5$	6.2	0.2		
F505A + mini-G_q	7.6	0.4	0.3	ns
F505A + mini-$G_q\Delta\alpha5$	7.1	0.3		
M508 + mini-G_q	7.8	0.2	0.0007	***
M508 + mini-$G_q\Delta\alpha5$	10.2	0.4		
W631A + mini-G_q	8.5	0.4	0.4	ns
W631A + mini-$G_q\Delta\alpha5$	9.5	1.0		
Q656A + mini-G_q	7.0	0.3	0.2	ns
Q656A + mini-$G_q\Delta\alpha5$	7.7	0.5		
F625A + mini-G_q	10.3	0.8	0.007	**
F625A + mini-$G_q\Delta\alpha5$	7.4	0.3		
L626A + mini-G_q	10.5	0.7	0.004	**
L626A + mini-$G_q\Delta\alpha5$	7.4	0.4		
L627A + mini-G_q	8.8	0.9	0.5	ns
L627A + mini-$G_q\Delta\alpha5$	8.0	0.5		
G628A + mini-G_q	8.1	0.6	1.0	ns
G628A + mini-$G_q\Delta\alpha5$	8.1	1.0		
H514A + mini-G_q	10.8	0.5	<0.0001	****
H514A + mini-$G_q\Delta\alpha5$	6.8	0.3		
L515A + mini-G_q	6.0	0.5	0.04	*
L515A + mini-$G_q\Delta\alpha5$	4.7	0.2		
Y516A + mini-G	8.2	0.7	0.4	ns
Y516A + mini-$G_q\Delta\alpha5$	7.5	0.3		

**Proximity assay comparisons**	**Normalised luminescence**	**SEM**	***p*-value**	**level of significance**
CTF1 + mini-G_q	18.28	1.0	<0.0001	****

F505A + mini-G_q	7.6	0.4		
CTF1 + mini-G_q	18.28	1.0	<0.0001	****
M508 + mini-G_q	7.8	0.2		
CTF1 + mini-G_q	18.28	1.0	<0.0001	****
W631A + mini-G_q	8.5	0.4		
CTF1 + mini-G_q	18.28	1.0	<0.0001	****
Q656A + mini-G_q	7.0	0.3		
CTF1 + mini-G_q	18.28	1.0	0.0001	***
F625A + mini-G_q	10.3	0.8		
CTF1 + mini-G_q	18.28	1.0	0.0001	***
L626A + mini-G_q	10.5	0.7		
CTF1 + mini-G_q	18.28	1.0	<0.0001	****
L627A + mini-G_q	8.8	0.9		
CTF1 + mini-G_q	18.28	1.0	<0.0001	****
G628A + mini-G_q	8.1	0.6		
CTF1 + mini-G_q	18.28	1.0	<0.0001	****
H514A + mini-G_q	10.8	0.5		
CTF1 + mini-G_q	18.28	1.0	<0.0001	****
L515A + mini-G_q	6.0	0.5		
CTF1 + mini-G_q	18.28	1.0	<0.0001	****
Y516A + mini-G	8.2	0.7		

Supplementary Table 4 | **7TM core alanine mutagenesis mini-G_q coupling results.**

Supplementary Figures:

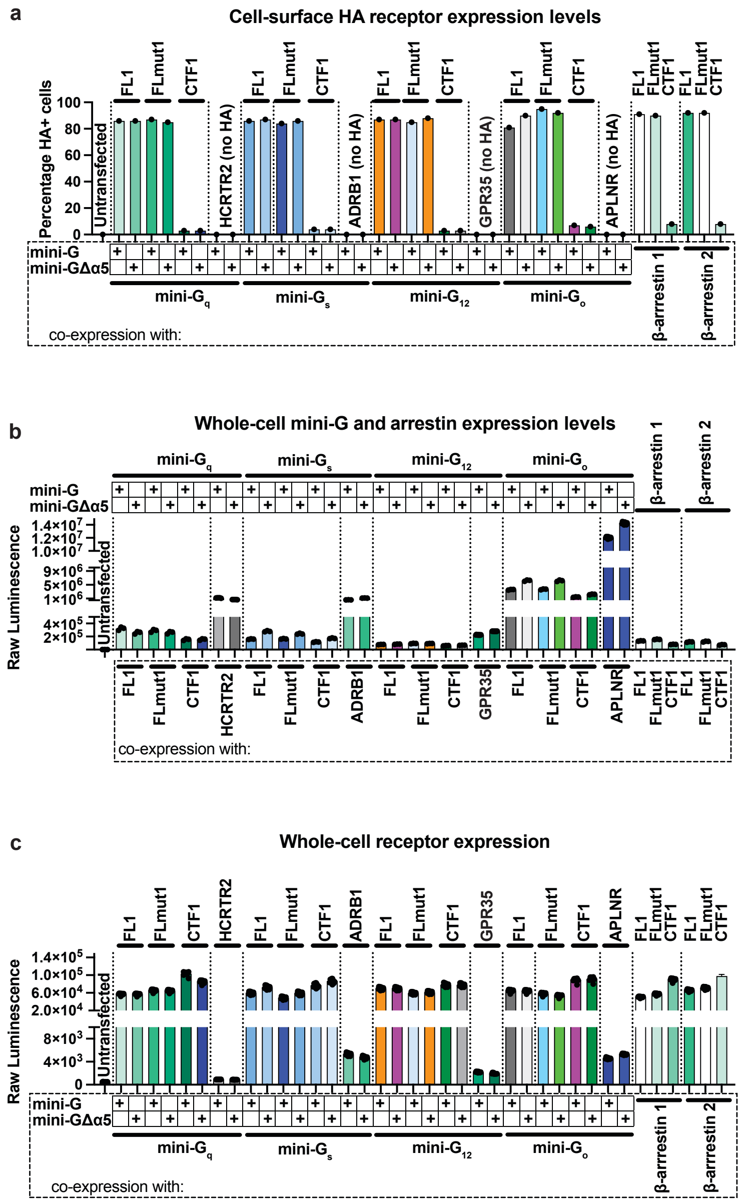

Supplementary Fig. 1 | **Representative receptor-SmBiT and LgBiT-mini-G/β-arrestin expression levels (n=3). a**, HA-tag cell surface expression levels as detected by flow cytometry. Constructs used as positive controls did not contain an HA-tag (HCRTR2, ADRB1, GPR35, APLNR). **b**, Whole cell expression of LgBiT-tethered mini-G/β-arrestin constructs as detected using Nano-Glo. **c,** Whole-cell expression of SmBiT-tethered G protein coupling constructs as detected using Nano-Glo. All ADGRL4 coupling constructs had similar whole-cell expression levels. The observed differences in whole-cell expression levels between positive controls and ADGRL4 constructs was due to the use of distinct promoters: the positive control receptors were expressed under a weak thymidine kinase (TK) promoter, whereas the ADGRL4 constructs were expressed under a strong cytomegalovirus (CMV) promoter.

Although the above displays results from a single representative biological replicate, all mini-G_q, mini-G_s, mini-G_{12}, mini-G_o and β–arrestin related assays (cell-surface HA flow cytometry, whole-cell mini-G levels, whole-cell receptor expression) were completed as three biological replicates, with each assay containing 3 technical replicates. All data values are reported in the source data file.

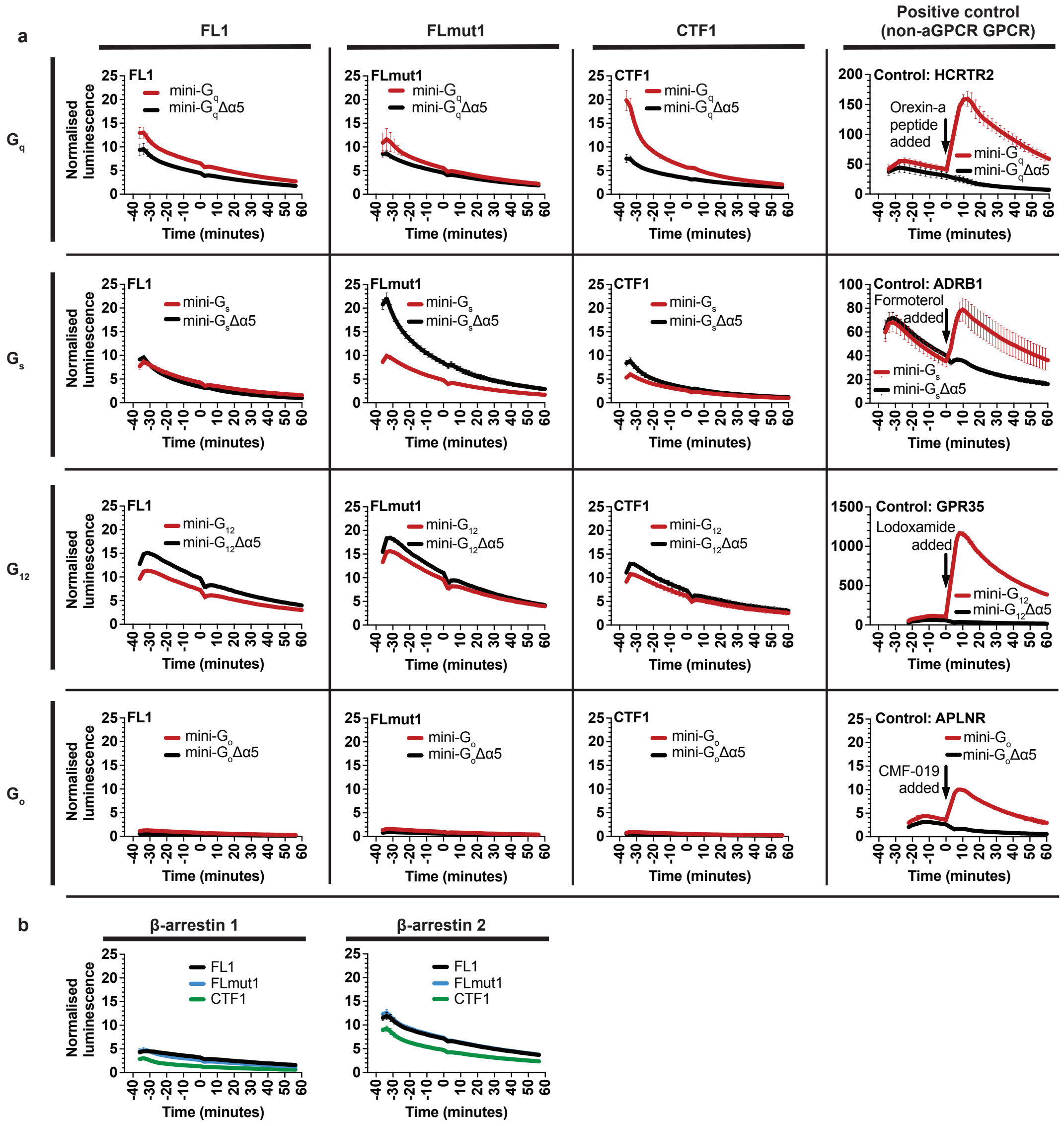

Supplementary Fig. 2 | **Representative complementation assays. a**, Representative mini-G complementation assays (n=3) showing that ADGRL4-CTF1 couples weakly to G_q. There was no increase in luciferase activity to suggest coupling to G_s, G_{12}, or G_o (n=3). **b**, Negative representative β-arrestin complementation assays (n=3).

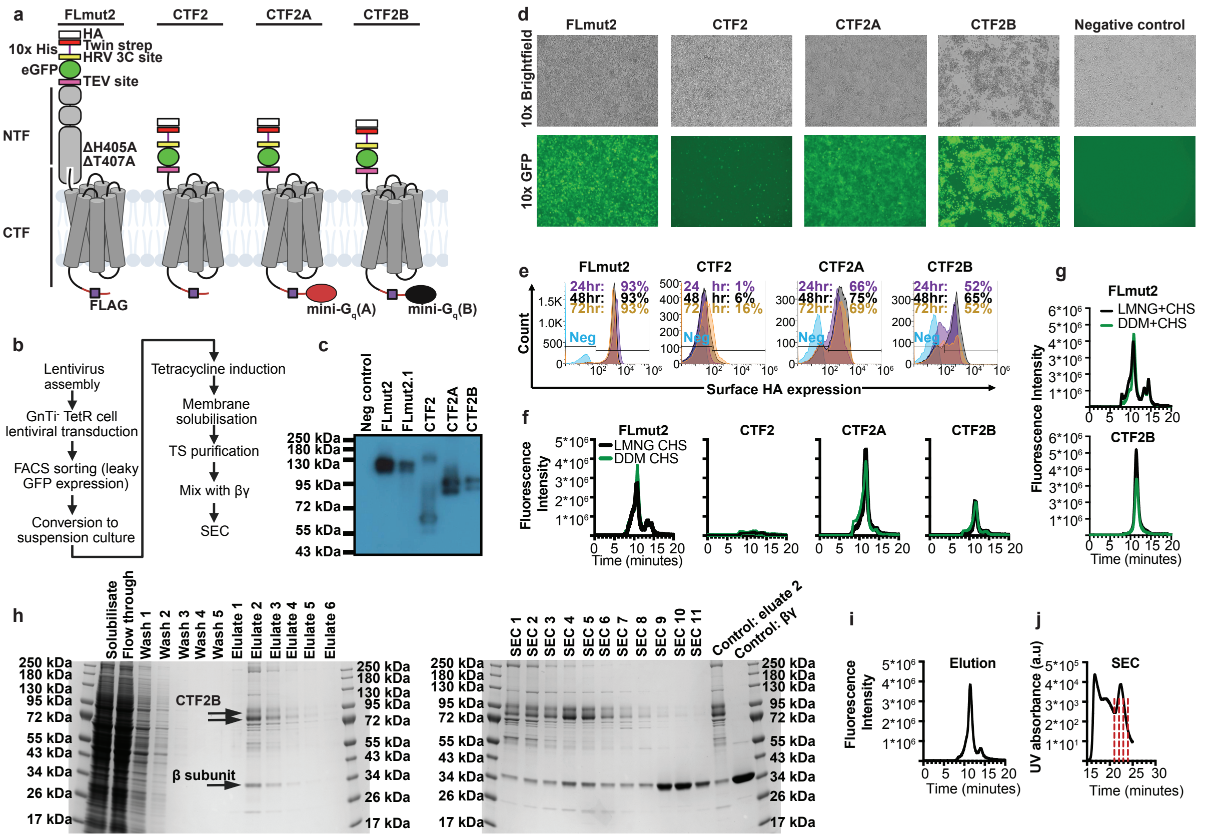

Supplementary Fig. 3 | **The design, expression and purification of ADGRL4 constructs for structural studies. a**, Cartoon representation of ADGRL4 constructs for structural studies, highlighting N-terminal and C-terminal tags and modifications. Mini-G_q (version A) and mini-G_q (version B) were C-terminally tethered to the receptor in constructs ADGRL4-CTF2A and ADGRL4-CTF2B respectively: version A is unable to bind βγ after binding to the intracellular portion of its cognate GPCR, whilst version B can. **b**, Summary of the overexpression and purification strategy. **c**, Western blot probed with an anti-HA antibody of ADGRL4 constructs expressed in adherent HEK293 GnTI$^-$ TetR stable cell lines. ADGRL4-FLmut2.1 refers to an alternative ADGRL4-FLmut2 variant that was not used further due to low expression. The predicted molecular weights for the assayed ADGRL4 constructs were: ~140kDa for FLmut2 and FLmut2.1; ~74kDa for CTF2; ~100kDa for CTF2A; and ~103kDa for CTF2B. **d**, Brightfield and fluorescent microscopy images of adherent HEK293 GnTI$^-$ TetR stable cell lines overexpressing ADGRL4 constructs after 72 hours of tetracycline induction. Overexpression of ADGRL4-CTF2B led to significant cell death and a pronounced loss of cellular adherence compared to other constructs. **e**, Flow cytometry time-course of ADGRL4 constructs showing cell-surface expression levels probed with an anti-HA antibody in adherent cells. ADGRL4-FLmut2 had the highest level of surface expression, whilst ADGRL4-CTF2 had the lowest. The C-terminal addition of mini-G_q (version A or B) to ADGRL4-CTF2A and ADGRL4-CTF2B substantially improved cell surface expression. **f**, FSEC trace of membrane solubilised

ADGRL4 constructs (small-scale solubilisation from adherent tetracycline-induced stable cell lines) showing similar peaks for each construct treated with different detergents (LMNG+CHS or DDM+CHS). No FSEC peak was detectable for ADGRL4-CTF2. **g**, FSEC trace of membrane solubilised ADGRL4 constructs (large-scale solubilisation from suspension tetracycline-induced stable cell lines). **h**, Coomassie stained SDS-PAGE gel analysis of purified ADGRL4-CTF2B. Eluate 2 was concentrated and then mixed with βγ prior to SEC. SEC fractions 4-6 were collected for cryo-EM studies. Arrows point to ADGRL4-CTF2B and GNB1 (G protein subunit β1) in eluate 2 as detected by mass spectrometry. **i**, FSEC trace of eluted ADGRL4-CTF2B. **j**, SEC trace (A280nm) of ADGRL4-CTF2B after mixing with βγ. The red lines indicate fractions collected for cryo-EM studies. (Abbreviations: FSEC= fluorescence-detection size-exclusion chromatography; GFP=green fluorescence, TS=twin strep).

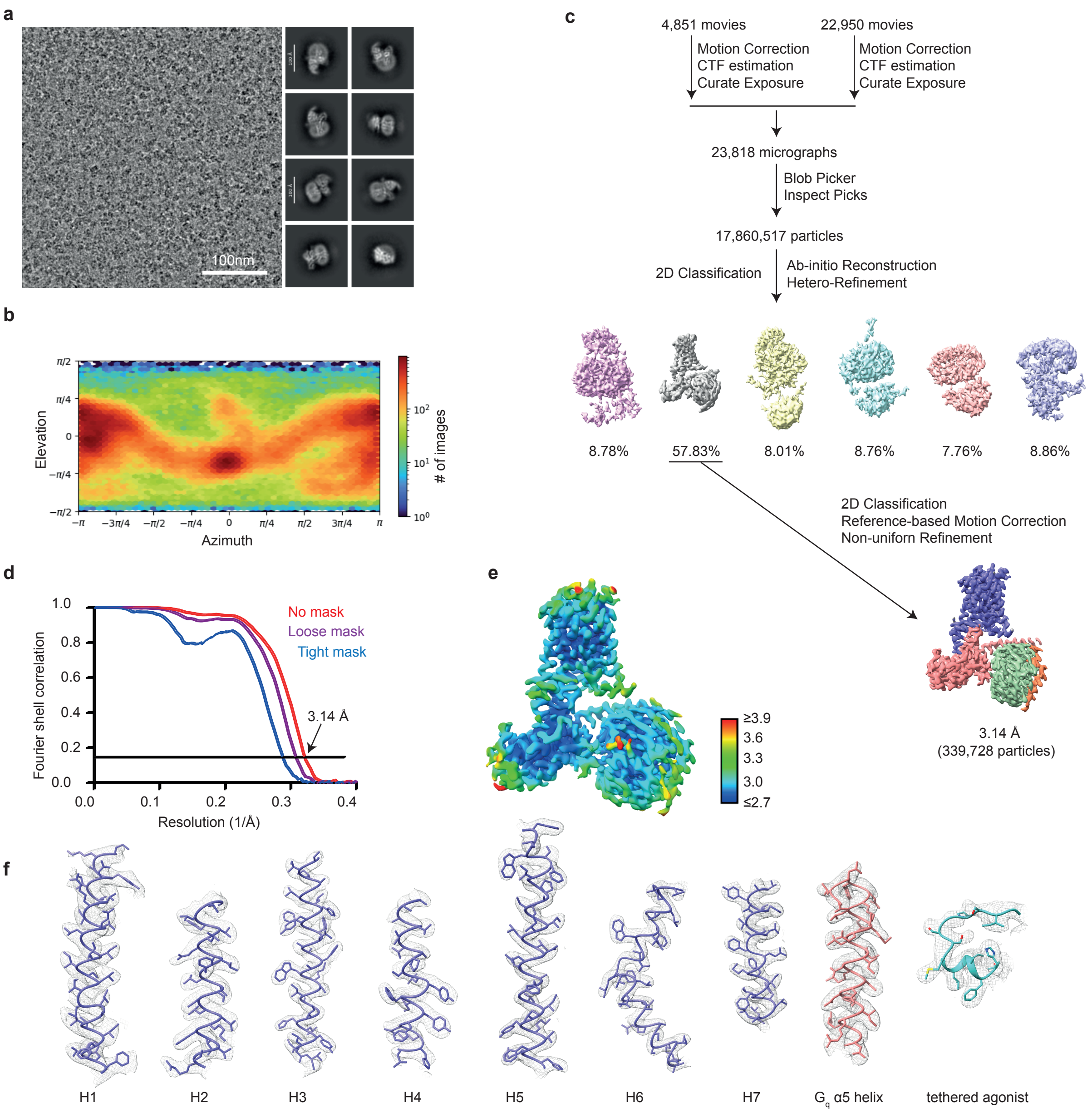

Supplementary Fig. 4 | **Active-state ADGRL4-CTF2B/βγ complex cryo-EM processing. a**, Representative cryo-EM micrograph and reference free 2D class averages of active-state ADGRL4-CTF2B/βγ complex. **b**, The angular distribution heat map of active-state ADGRL4-CTF2B/βγ complex showing particle projections. **c**, Cryo-EM data processing workflow for active-state ADGRL4-CTF2B/βγ complex. **d**, Fourier shell correlation (FSC) curve for the ADGRL4-CTF2B/βγ complex density. The overall nominal resolution was 3.1 Å using the gold standard FSC = 0.143. **e**, Local resolution cryo-EM map for active-state ADGRL4-CTF2B/βγ complex. **f**, Cryo-EM density maps for transmembrane region α-helices H1-H7 in ADGRL4-CTF2B, G_q α5 helix and the bound tethered agonist.

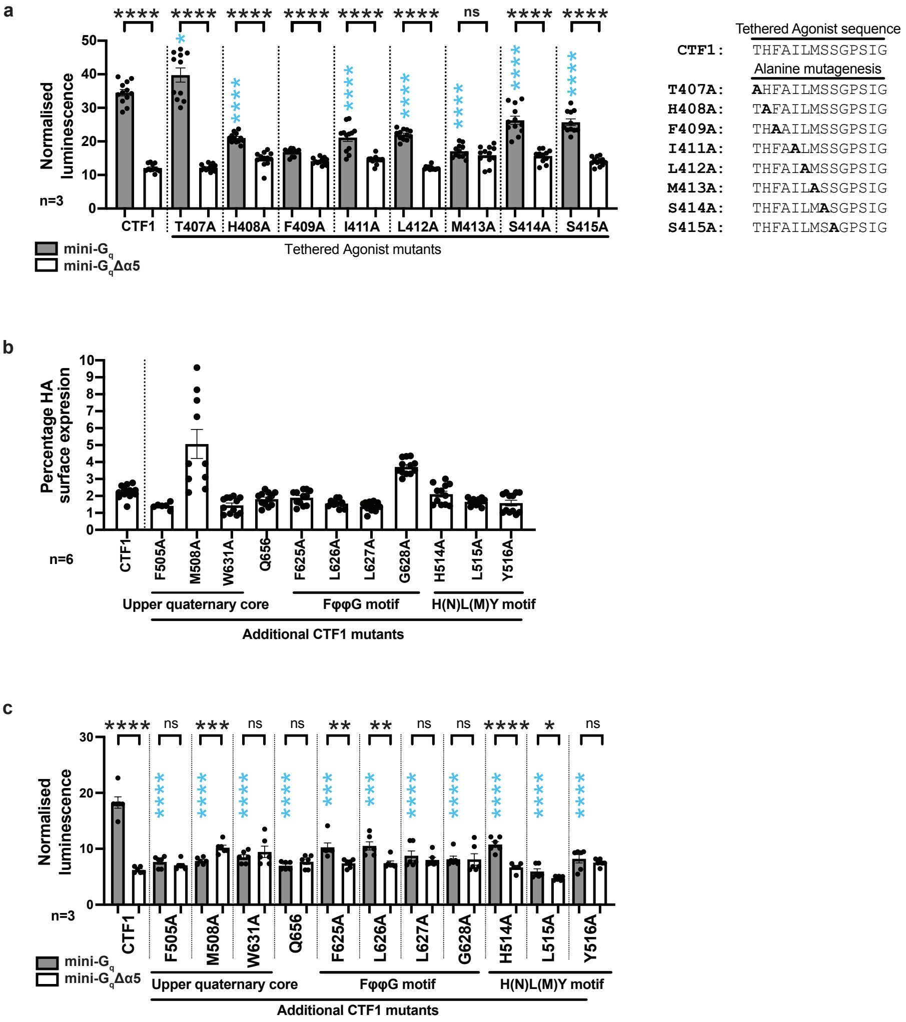

Supplementary Fig. 5 | **Expanded tethered agonist and 7TM activation motifs alanine mutagenesis G_q complementation assays. a**, Interactions between tethered agonist mutagenesis constructs (SmBiT tagged) and LgBiT-mini-G_q or LgBiT-mini-$G_q\Delta\alpha5$ constructs were determined by luminescence. The blue stars refer to comparisons to CTF1 + mini-G_q. Data represent three biological replicates, with each assay containing four technical replicates; error bars represent the SEM (****, p<0.0001; *, p<0.05; ns, non-significant). All data values and significance values are listed in Supplementary Table 3. **b**, Cell surface expression levels of 7TM activation motif mutagenesis constructs contrasted with CTF1, as determined by flow cytometry (α-HA). Data represent six biological replicates performed in duplicate; error bars represent the SEM. **c**, Interactions between 7TM activation motif mutagenesis constructs (SmBiT tagged) and LgBiT-mini-G_q or LgBiT-mini-$G_q\Delta\alpha5$ constructs as determined by luminescence. The blue stars refer to comparisons to CTF1 + mini-G_q. Data represent three biological replicates, with each assay containing two technical replicates; error bars represent the SEM (****, p<0.0001; ***, p<0.001; **, p<0.01; p<0.05; ns, non-significant). All data values and significance values are listed in Supplementary Table 3.

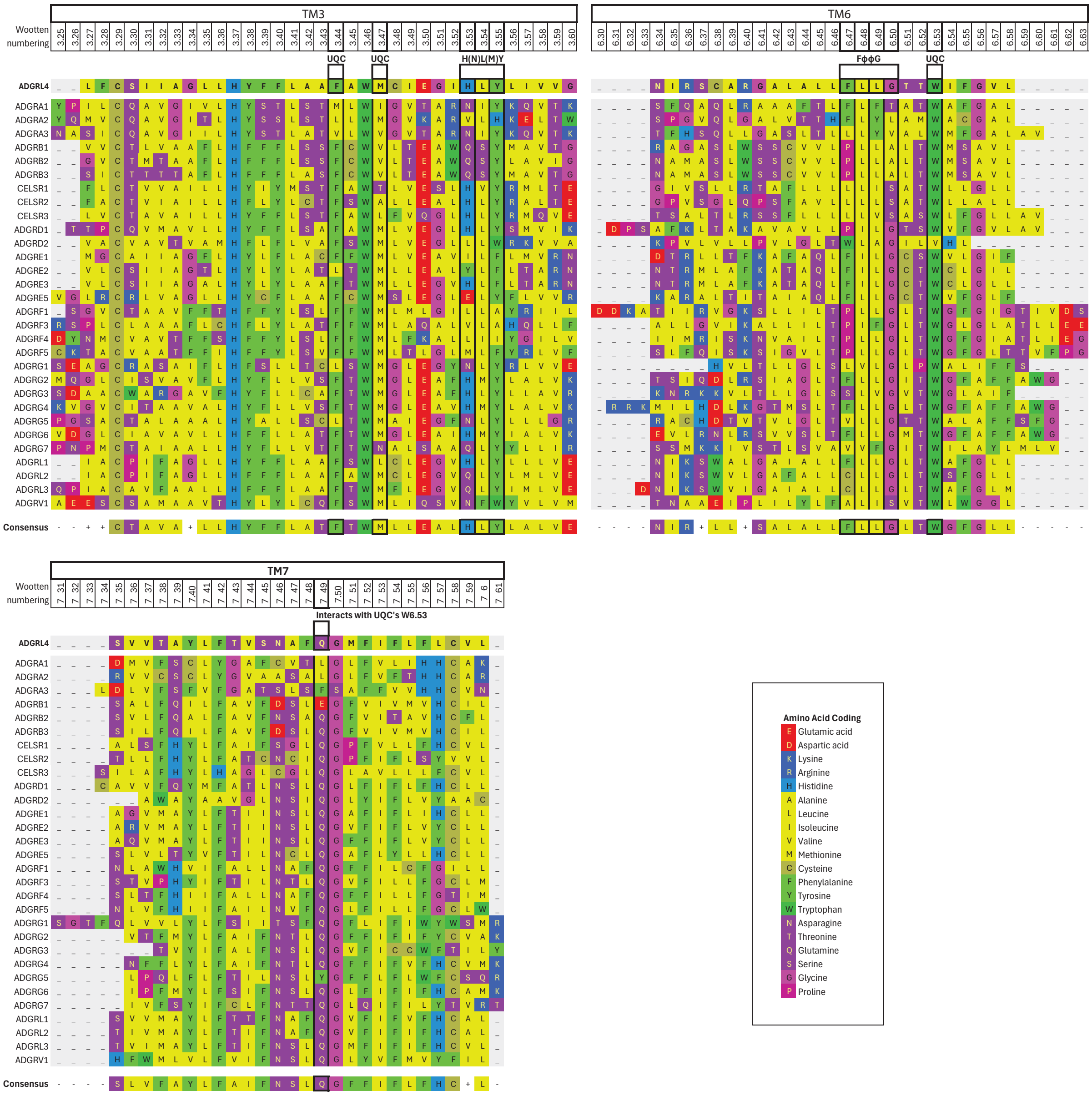

Supplementary Fig. 6 | **Expanded aGPCR amino acid alignment.** Alignment of 31 human protein-coding aGPCRs showing transmembrane loops 3, 5 and 7, with Wootten numbering[1]. 7TM activation motif regions for ADGRL4 are highlighted with white boxes: upper quaternary core (UQC), FφφG and H(N)L(M)Y motifs (φ refers to hydrophobic residues). ADGRF2 and ADGRE4P were not included in the alignment as both human genes are considered to be pseudogenes.

Supplementary Raw Data:

a

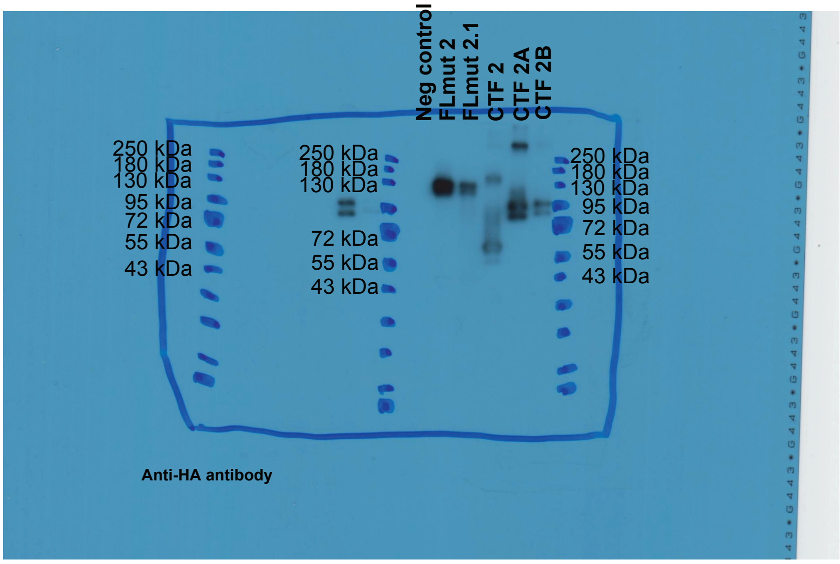

b

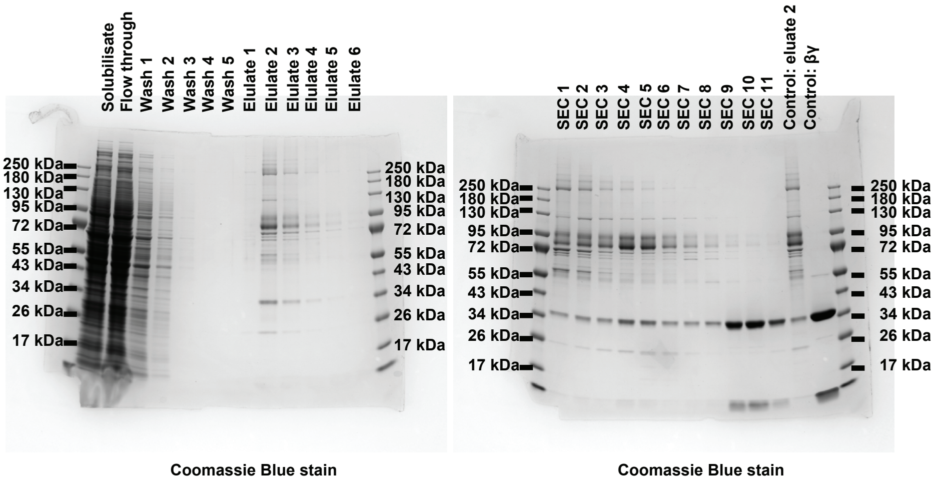

Raw data. Raw western blot X-ray film (**a**) used in Supplementary Fig. 3C, and SDS-PAGE gel (**b**) used in Supplementary Fig. 3H.

Supplementary References:

1 Wootten, D., Simms, J., Miller, L. J., Christopoulos, A. & Sexton, P. M. Polar transmembrane interactions drive formation of ligand-specific and signal pathway-biased family B G protein-coupled receptor conformations. *Proceedings of the National Academy of Sciences of the United States of America* **110**, 5211-5216 (2013). https://doi.org/10.1073/pnas.1221585110
